# Calibration of the Relative Orientation between Multiple Depth Cameras Based on a Three-Dimensional Target

**DOI:** 10.3390/s19133008

**Published:** 2019-07-08

**Authors:** Zhe Liu, Zhaozong Meng, Nan Gao, Zonghua Zhang

**Affiliations:** 1 State Key Laboratory of Reliability and Intelligence of Electrical Equipment, Hebei University of Technology, Tianjin 300130, China; 2 School of Mechanical Engineering, Hebei University of Technology, Tianjin 300130, China

**Keywords:** three-dimensional target, lidar, orientation calibration, multiple depth cameras

## Abstract

Depth cameras play a vital role in three-dimensional (3D) shape reconstruction, machine vision, augmented/virtual reality and other visual information-related fields. However, a single depth camera cannot obtain complete information about an object by itself due to the limitation of the camera’s field of view. Multiple depth cameras can solve this problem by acquiring depth information from different viewpoints. In order to do so, they need to be calibrated to be able to accurately obtain the complete 3D information. However, traditional chessboard-based planar targets are not well suited for calibrating the relative orientations between multiple depth cameras, because the coordinates of different depth cameras need to be unified into a single coordinate system, and the multiple camera systems with a specific angle have a very small overlapping field of view. In this paper, we propose a 3D target-based multiple depth camera calibration method. Each plane of the 3D target is used to calibrate an independent depth camera. All planes of the 3D target are unified into a single coordinate system, which means the feature points on the calibration plane are also in one unified coordinate system. Using this 3D target, multiple depth cameras can be calibrated simultaneously. In this paper, a method of precise calibration using lidar is proposed. This method is not only applicable to the 3D target designed for the purposes of this paper, but it can also be applied to all 3D calibration objects consisting of planar chessboards. This method can significantly reduce the calibration error compared with traditional camera calibration methods. In addition, in order to reduce the influence of the infrared transmitter of the depth camera and improve its calibration accuracy, the calibration process of the depth camera is optimized. A series of calibration experiments were carried out, and the experimental results demonstrated the reliability and effectiveness of the proposed method.

## 1. Introduction

In recent years, the number of depth camera applications in our daily lives has increased dramatically with the reduction of equipment cost [1]. Depth cameras play an important role in machine vision-related fields, such as human face recognition [2,3], three-dimensional scene reconstruction [4], and limb motion capture [5]. Due to the diversification of application requirements, higher demands are put forward for 3D information acquisition, such as more precise scanning of scenes and more rapid modeling of objects. A single depth camera no longer satisfies the practical requirements of some application scenarios. Multiple depth camera systems can potentially solve the above problems with accuracy and speed, and offer outstanding advantages compared to single depth cameras.

In order to apply multiple depth camera systems to different scenes and ensure the accuracy and efficiency of measurements, we need to calibrate these systems to obtain the key parameters. In recent years, many scholars have conducted investigations on camera system calibration, and have achieved remarkable progress [6,7,8,9,10,11,12,13,14,15]. Among them, the active vision-based camera calibration method allows the calculation of the internal camera parameters without a calibration object, with only performing specific motions with the camera [6]. The advantage of this method is its simple algorithm and robustness, but the difficulty of its application in multiple depth camera systems is higher due to the high cost of the system and the requirement of experimental setup. Traditional camera calibration methods need to use calibration plates with known dimensions. By establishing the corresponding relationship between feature points with known coordinates on the calibration plates and their pixel points, the relationship between the three-dimensional coordinates and the two-dimensional plane coordinates can be obtained, and the internal and external parameters of the camera model can be obtained using certain algorithms [7,8]. In addition, the most classical camera calibration method is to use plane targets for the calibration, as proposed by Zhang Z. [12]. This method uses a camera to capture a chessboard pattern in different directions to achieve calibration, which is easy to perform and provides high accuracy. Therefore, it is widely used for single camera calibration. Since the measurement range of the planar target is limited to one feature surface, and multiple depth camera systems have large viewing angles, the planar target method is not suitable for calibrating multiple depth camera systems. The merging of multiple depth camera systems with multiple coordinate systems into a single coordinate system and calibrating without overlapping fields of view is an important research topic. Thus, the integration of the coordinate system of multiple depth cameras based on their view field is the key issue for their calibration. The calibration method proposed by Manuel et al. can be defined as a hybrid solution, consisting of photogrammetry and self-calibration methods [13], which needs special markers for the calibration. Pedersini et al. used two overlapping planar calibration boards to calibrate the multiple camera systems [14]. Their method is based on a self-calibration approach, which allows the refinement of the prior knowledge of the world coordinates of the targets while estimating the parameters of the camera model. Shen et al. presented a complete calibration methodology using a non-planar calibration target with a sphere for rapid calibration [15] based on the precise fitting of ellipses. Avetisyan et al. presented a simple method for calibrating multiple cameras reliably using a tracking system with a trackable calibration target [16]. Beck et al. realized a method for the calibration and registration of multiple RGBD sensors by sweeping a tracked checkerboard through the capturing space in front of each sensor [17,18]. All methods require a tracked calibration target to eliminate the problems caused by the needs for overlapping views. However, the calibration process of these methods relies heavily on the manufacturing accuracy of calibration target. Huang et al. designed a 3D calibration object to calibrate multiple camera systems [19]. This method does not calibrate the 3D calibration object, but only relies on ideal world coordinates for subsequent calibration, and its error is difficult to measure. Current calibration methods have many limitations, such as complex algorithms, poor operability, vulnerability to the influence of multi-camera fields of view and other factors and are thus inapplicable for actual products to a satisfactory degree.

In addition, scholars have proposed many improved methods for the calibration of depth cameras [20]. The functionality of popular depth cameras on the market is mainly accomplished through infrared imaging and projectors, hence the depth image is “carried” by the infrared image [21]. So, in essence, the calibration of depth cameras is equivalent to calibrating an infrared camera. Herrera et al. proposed a calibration model of depth camera parameters based on the original disparity of the camera [22], and this method can obtain more accurate results through longer calculations. Smisek et al. proposed to calibrate infrared cameras by shielding the infrared projector to avoid the speckle effect [23], as the calibration error of depth cameras is mainly caused by the speckle of the infrared projector. This method is simple and easy to apply. However, current methods are time consuming for high accuracy results, or the results are unstable.

In this paper, a method for the calibration of multiple depth camera systems using a 3D target at specific angles is proposed. The specific angle of the three-dimensional target is determined by the combination of cameras in the multiple depth camera systems. This angle can ensure that multiple cameras can simultaneously capture chessboard images in full camera field of view for calibration. This 3D target allows the calibration of multiple depth cameras at relative orientations with limited or no overlapping fields of view. In addition, the point cloud is obtained by scanning the 3D target with a high-precision lidar. These point clouds are used to calibrate the 3D target to ensure that the position and posture of the calibration plate are accurate and reliable. Moreover, the calibration process of the depth camera is optimized. The results of a series of comparative experiments demonstrate that the method proposed in this paper is efficient and accurate.

The paper is organized as follows. In Section 2, a 3D calibration target is designed, a method for precise calibration using 3D targets and a lidar is presented and a pin-hole depth camera calibration model is introduced. In addition, a global calibration method for multiple depth cameras is proposed. In Section 3, the experiment results we obtained from the application of the proposed method are compared against well-known methods. Finally, Section 4 concludes the work.

## 2. Principle

In order to calibrate the relative orientation between multiple depth cameras simultaneously, a 3D target has been designed. It can be used to integrate the coordinates of each depth camera into one coordinate system quickly and effectively. Each calibration plate on the 3D target can calibrate a camera independently, so it can calibrate multiple cameras simultaneously.

### 2.1. Design of 3D Target

**Figure 1:**
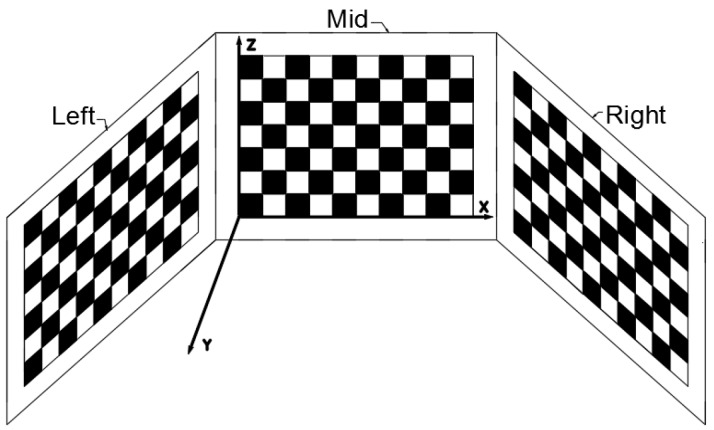
3D target model.

The 3D target is as shown in Figure 1, and consists of three planar calibration boards to ensure that each camera can capture enough calibration information.

The established coordinate system is shown in Figure 1, with the bottom of the middle calibration board as the origin of the unified coordinate. Since the location of each point is already known, the spatial coordinates of feature points can be easily obtained. The method for unifying the coordinates of the feature points on the 3D target to the same world coordinate system is described in Section 2.2.

**Figure 2:**
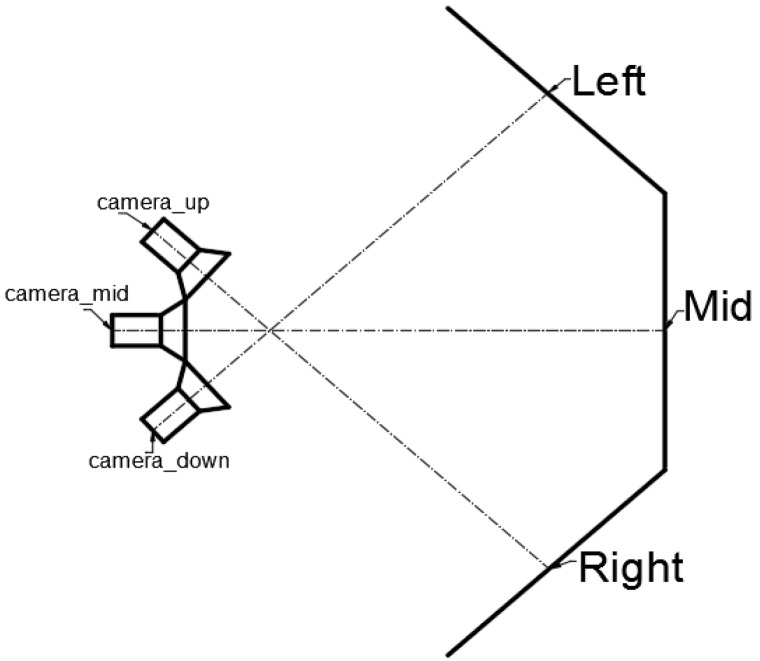
Structure of the designed multiple depth camera system.

Three depth cameras can be calibrated at one time by the 3D target. Three depth cameras are positioned directly opposite the stereo target, as shown in Figure 2. The distance between the camera and the 3D target is determined by the focal length of the camera and should be such that the calibration board covers the field of view of each camera as fully as possible, so that each camera can capture enough calibration information. Each camera must be able to obtain a clear image at this distance. Each camera corresponds to a calibration board and only needs to capture the corresponding board information; camera_up corresponds to the Right calibration board, camera_down corresponds to the Left calibration board and camera_mid corresponds to the Mid calibration board. As long as the calibration board is fully covered by the camera’s field of view, it the angle between the calibration boards does not matter. There are no other special requirements for the tolerance of the 3D calibration target, because a high precision lidar will be used to accurately calibrate the relative position of the 3D calibration target, as explained in Section 2.2. When calibrating the internal parameters of the cameras, Zhang’s plane calibration method can be used to obtain the camera’s internal parameters by slightly moving the camera to take some photos. When calibrating the external parameters of the camera, the only operation is to place the three depth cameras in front of the 3D target and obtain an image. Because the coordinates of the feature points are obtained from the same coordinate system, the three depth camera coordinate systems can be unified into a single system using three photos, thus the spatial position of the cameras can be finally obtained.

### 2.2. Calibration of the 3D Target

In this part, the calibration using the 3D target and the unification of the coordinate systems of the feature points are introduced in detail.

The calibration of the 3D target is the basis of the whole calibration process. Accurate calibration of the 3D target can reduce the error in the subsequent calibration process effectively. The point cloud is obtained by scanning the 3D target with a high precision lidar, and the point cloud is used to calibrate the target.

Firstly, the plane of the point cloud should be fitted. The plane equation can be described as:

(1) xcos∝+ycosβ+zcosγ+p=0,

where (cos∝, cosβ, cosγ) is the normal vector direction cosine at point (x, y, z) on the plane. ∣p∣ is the distance from the origin to the plane.

The distance from any data point to the plane is:

(2) $$d_{i}=|ax_{i}+by_{i}+cz_{i}-d\;|,i=1,\ldots ,N,$$

In order to obtain the best fitting plane, Equation (3)’s value needs to be normalized:

(3) $$f=\overset{n}{\underset{i=0}{\sum }}d_{i}{}^{2}-k\left(a^{2}+b^{2}+c^{2}-1\right),$$

Equation (4) can be obtained by calculating the partial derivatives of f for d, a, b and c respectively:

(4) $$\left[\begin{array}{ccc} \sum^{\;}\Delta x_{i}\Delta x_{i} & \sum^{\;}\Delta x_{i}\Delta y_{i} & \sum^{\;}\Delta x_{i}\Delta z_{i}\\ \sum^{\;}\Delta x_{i}\Delta y_{i} & \sum^{\;}\Delta y_{i}\Delta y_{i} & \sum^{\;}\Delta y\Delta z_{i}\\ \sum^{\;}\Delta x_{i}\Delta z_{i} & \sum^{\;}\Delta y_{i}\Delta z_{i} & \sum^{\;}\Delta z_{i}\Delta z_{i} \end{array}\right]\left[\begin{array}{c} a\\ b\\ c \end{array}\right]=k\left[\begin{array}{c} a\\ b\\ c \end{array}\right],$$

Therefore, the normal vector of the plane can be expressed as:

(5) n=(a,b,c),

After obtaining the normal vectors of the three calibration plates, it is necessary to unify the three planes into the laser scanner’s coordinate system. If we define the normal vector $n_{0}=\left(0,0,1\right)$, the rotation angle from each plane to the laser scanner is:

(6) $$\theta_{left}=\arccos\frac{n_{left}\cdot n_{0}}{|n_{left}|\cdot |n_{0}|},$$

The plane where the rotation angle is located is composed of vectors $n_{left}$ and $n_{0}$. Then, the axis of rotation must be perpendicular to that plane according to the cross-product equation:

(7) $$n_{left}\times n_{0}=\left|\begin{array}{ccc} i & j & k\\ A_{left} & B_{left} & C_{left}\\ A_{0} & B_{0} & C_{0} \end{array}\right|,$$

where **i**, **j**, **k** are the unit vectors of the x, y, z axes respectively.

So, the rotation axis $c_{left}\left(c_{1},c_{2},c_{3}\right)$ is:

(8) $$\left[\begin{array}{c} c_{1}\\ c_{2}\\ c_{3} \end{array}\right]=\left[\begin{array}{c} B_{left}C_{0}-C_{left}B_{0}\\ C_{left}A_{0}-A_{left}C_{0}\\ A_{left}B_{0}-B_{left}A_{0} \end{array}\right],$$

The unit vector of the rotation axis is obtained as follows:

(9) $$c_{left}^{\prime }=\frac{c_{left}}{|c_{left}|}=\left[\begin{array}{c} c_{1}^{\prime }\\ c_{2}^{\prime }\\ c_{3}^{\prime } \end{array}\right],$$

Given the rotation axis and its corresponding rotation angle, the rotation matrix can be obtained according to Rodrigues’ rotation equation [24]:

(10) $$R_{left}=I+\omega_{left}\sin\theta_{left}+\omega_{left}{}^{2}\left(1-\cos\theta_{left}\right),$$

with

(11) $$\omega_{left}=\left[\begin{array}{ccc} 0 & -c_{3}^{\prime } & c_{2}^{\prime }\\ c_{3}^{\prime } & 0 & -c_{1}^{\prime }\\ -c_{2}^{\prime } & c_{1}^{\prime } & 0 \end{array}\right],$$

where **I** is the 3×3 unit matrix, and $\omega_{left}$ denotes the "cross-product matrix" for the unit vector $c_{left}^{\prime }$. Similarly, we obtain the rotation matrices $R_{mid},R_{right}$.

After rotating the plane using the rotating matrix, the translation matrix can be obtained by calculating the distance between two points. Before the rotation, we first fit the plane, and we can decipher that the center point of the best fitting plane is $p_{left}$. After the rotation, the plane is transferred to the coordinate system of the laser scanner, so we choose point $p_{0}=\left(0,0,0\right)$. And the translation matrix is:

(12) $$T_{left}=n_{0}\cdot (R_{left}p_{left}^{T}-p_{0}^{T}\;),$$

similarly, we can obtain the translation matrix $T_{mid},T_{right}$.

So far, the rotation and translation matrices of the three planar calibration targets have been obtained. The feature points’ coordinates of the 3D target are unified into the same world coordinate system simultaneously.

### 2.3. Calibration of Multiple Depth Cameras

In this section, the calibration of three depth cameras using the 3D target is introduced. The depth camera consists of an infrared camera and an infrared projector. Thus, depth information is contained in infrared images. Because the infrared projector will interfere with the image taken by the infrared camera, the infrared projector is blocked during calibration. In order to ensure stable calibration results, halogen lamps are used to increase ambient brightness and to obtain proper infrared images.

The infrared camera is suitable for the pin-hole model, including radial correction and tangential correction. According to Zhang’s calibration method, the transformation relationship between the camera coordinate system and world coordinate system is:

(13) $$s\left[\begin{array}{c} u\\ v\\ 1 \end{array}\right]=A\left[\begin{array}{cc} R & T \end{array}\right]\left[\begin{array}{c} X_{w}\\ Y_{w}\\ Z_{w}\\ 1 \end{array}\right],$$

with

(14) $$A=\left[\begin{array}{ccc} \alpha & \gamma & u_{0}\\ 0 & \beta & v_{0}\\ 0 & 0 & 1 \end{array}\right],$$

where s is an arbitary scale factor, (u,v) is the coordinate of a point in the image coordinate system, A is called the camera’s intrinsic matrix, $\left(u_{0},v_{0}\right)$ represents the coordinate of the principal point, α and β represent the scale factors in the image’s u and v axes, and γ is the parameter describing the skew of the two image axes. Normally, the default value of γ is 0. R is the rotation matrix and T is the translation matrix which are called the extrinsic parameters. $X_{w},\;Y_{w},Z_{w}$ are the world coordinates.

In Section 2.2, the coordinates of the feature points on the 3D target $X_{w},\;Y_{w},Z_{w}$ were unified into the same world coordinate system. The corresponding u and v coordinates can be obtained by processing the calibrated image. By moving the camera to obtain several sets of photos, the corresponding internal parameters can be obtained. The *R* and *T* of each depth camera relative to the calibration board can also be obtained. The relative position relationship between depth cameras can be obtained from the following steps. Firstly, *R* and *T* from the Left calibration board to the Mid calibration board can be expressed as:

(15) $$\{\begin{array}{l} R_{Left-Mid}=R_{Left}^{-1}R_{Mid}\\ T_{Left-Mid}=R_{Left}^{-1}\left(T_{Mid}-T_{Left}\right) \end{array},$$

Secondly, *R* and *T* from camera_down to Mid calibration board can be expressed as:

(16) $$\{\begin{array}{l} R_{down-Mid}=R_{down}R_{Left-Mid}\\ T_{down-Mid}=R_{Left-Mid}^{-1}\left(T_{Left-Mid}-R_{down}^{-1}T_{down}\right) \end{array},$$

Finally, the transformation relation between camera_mid and camera_down is described in Equation (17):

(17) $$\{\begin{array}{l} R_{mid-down}=R_{mid}R_{down-Mid}^{-1}\\ T_{mid-down}=R_{mid}T_{down-Mid}+T_{mid} \end{array},$$

Similarly, the transformation relation between camera_mid and camera_up can be obtained. The rotation matrix can be decomposed into:

(18) $$R=R_{x}R_{y}R_{z},$$

where $R_{x},R_{y},R_{z}$ are the rotation matrixes that rotate around the x, y and z axes, respectively. $R_{x}$ contains the angles between the cameras.

## 3. Experiments

### 3.1. Hardware Setup of Multiple Depth Camears

**Figure 3:**
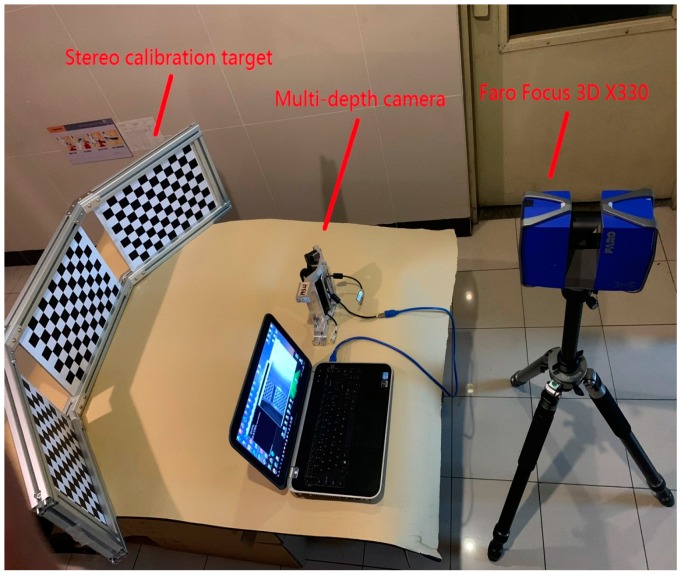
Multiple depth camera systems.

In order to verify the effectiveness of the proposed method, a multiple depth camera system was designed as shown in Figure 3. The system consisted of a 3D target with three planar chessboards combined at a specific angle, three ORBBEC Astra infrared cameras with a pixel resolution of 640 × 480 and a Faro Focus 3D X330 lidar with a distance accuracy of ±1 mm, which has very low noise. The lidar’s horizontal field of vision is 360° and its vertical field of vision is 300°. The vertical and horizontal step lengths of the lidar are both 0.09°. The cameras and Faro lidar follow the right-hand coordinate system. The experimental computer was a laptop with a CPU clock speed of 2.2 GHz, and the software environment was MATLAB R2015a.

The theoretical angle between the middle calibration board and the other two calibration boards was 138°, which is dependent on the angle between the multiple depth cameras. However, errors will be introduced in manufacturing and assembling, resulting in a deviation of the actual angle values. Our calibration using stereo targets solves these errors. Each planar chessboard had a size of 400 × 300 mm with 12 × 9 squares on it, and each square had a size of 30 × 30 mm. The Faro Focus 3D X330 lidar will automatically compensate during the scanning process to improve the accuracy.

**Figure 4:**
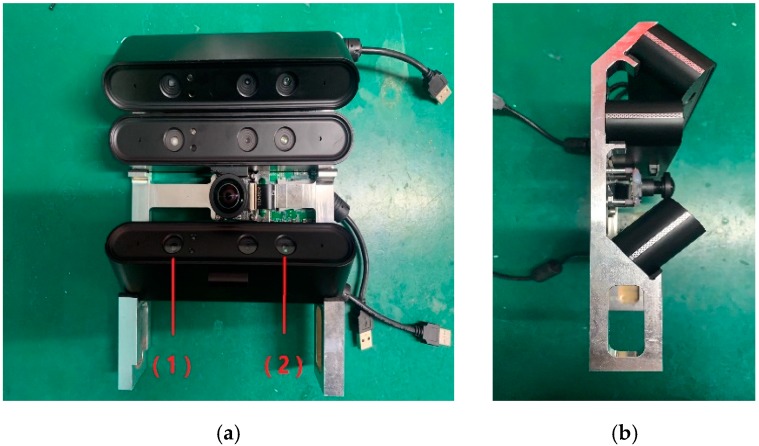
Placement of the multiple depth cameras. (**a**) and (**b**) are the front and side views, respectively. (1) is the infrared projector; (2) is the infrared camera.

The structure of the multiple depth cameras is shown in Figure 4. Each depth camera contains an infrared projector and an infrared camera. The theoretical value of the angle between each camera is 42°, but the actual value contains errors introduced during manufacturing and assembly, which require calibration to correct them.

### 3.2. Calibration

By placing the camera in a position where the 3D board covers the filled with field of view, the specific calibration steps are designed as follows:

1. Initialize the lidar and select medium scan quality. The 3D target is placed approximately 1 m away in front of the lidar. The point cloud data are processed to extract the calibration plate’s point cloud from the scanned point cloud. Calibration using the 3D target’s processed point cloud data and the *R*, *T* of each calibration board relative to the lidar is performed.
2. Block the infrared projector and use a halogen lamp to illuminate the 3D target. Move the camera around the 3D target repeatedly and ensure that images of the 3D target from different angles are obtained. Approximately 15 pictures are obtained.
3. The images are processed, and the coordinates of feature points are extracted for calibration. The internal parameters of the depth camera and the external parameters of each camera relative to the 3D target are obtained.
4. Each depth camera takes a picture of the corresponding calibration board filling its field of view. For example, camera_up photographs the Right calibration board. Process the captured image to obtain the *R*, *T* and angle between each depth camera, that is, the spatial relationship between each depth camera.

**Figure 5:**
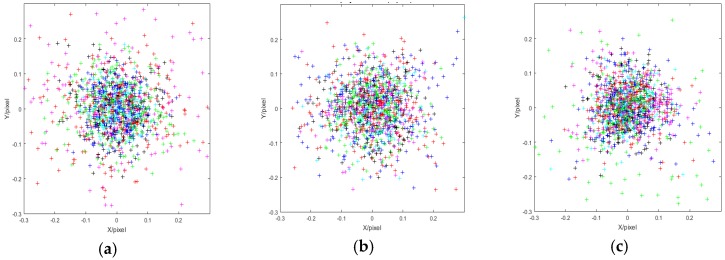
Distribution of the reprojection errors. (**a**), (**b**), and (**c**) are the reprojection errors of camera_up, camera_mid and camera_down, respectively.

The external parameters of each calibration board are given in Table 1, and the intrinsic parameters are given in Table 2, where $f_{x}$, $f_{y}$ are the equivalent focal length of the x and y axes, and $u_{0}$, $v_{0}$ are the principal points of the x and y axes, respectively. The external parameters of each camera are given in Table 3. The distribution of the reprojection error is as shown in Figure 5.

In Figure 5, each photograph contains the different reprojection errors of each camera at the fifteen locations. The errors are mainly distributed between −0.1 to 0.1 pixel in all three photographs. This demonstrates that the accuracy and stability of the method are both high.

### 3.3. Evaluation

In order to verify the effectiveness of the calibration method proposed in this paper, an additional experiment was carried out. Four common calibration methods were selected to compare with the method proposed in this paper. In method A, a single chessboard is used to calibrate the external parameters using Zhang Z.’s calibration method [12]. In method B, the external parameters are calibrated directly using an uncalibrated 3D target using Huang’s method [19]. In method C, the 3D target is scanned using a camera with known internal parameters, the relative pose of the 3D target is calculated, and the feature point coordinates of different coordinate systems are unified into the same coordinate system. Then, the external parameters of the multiple depth cameras are calculated. In method D, a method for the calibration and registration of multiple RGBD sensors by sweeping a tracked checkerboard through the desired capturing space in front of each sensor is used [17].

The feature point coordinates of the three planar calibration boards were unified on the same plane after calibration. Theoretically, the normal vector of this plane is ${\left(0,0,1\right)}^{T}$. Therefore, we simulated an ideal point cloud plane with a normal vector ${\left(0,0,-1\right)}^{T}$, which corresponds to the middle calibration board of the 3D target. The coordinates of the ideal plane with the normal vectors of ${\left(0,0,-1\right)}^{T}$ were unified using the rotation matrix of the middle calibration board obtained by the three calibration methods, respectively. The normal vectors of the two new planes obtained using the three calibration methods are shown in Table 4.

It is clear that the angle between the actual plane and the ideal plane is significantly reduced when the laser is used to calibrate the 3D target. This means that our calibration method has a smaller than the other two methods, and there has been a significant improvement with respect to accuracy.

Then, further experimental comparisons on the calibration using the 3D target with different calibration methods were carried out. Five methods were applied to calibrate the multiple depth camera system. The angle between the cameras for the five methods is given in Table 5 and the RMS value of reprojection errors of the five calibration methods are given in Table 6.

As shown in Table 5, the single chessboard used in method A is limited by the field of view coincidence of the multi-camera system, which results in very large error in the calibration results, and the results fluctuate greatly in repeated calibrations. The 3D target used in method B is limited by the manufacturing accuracy of the device, and the calibration result error is large. In method C, although the relative pose of 3D target has been taken into account, the accuracy of the camera is limited, and the result is less accurate. The accuracy of calibration results is further improved in method D. The angle between the cameras obtained using the method proposed in this paper is very close to the ideal angle which shows that this method results in a high accuracy for calibrating the external parameters of multiple depth camera systems. As shown in Table 6, the method proposed in this paper greatly reduces RMS error which means our method has higher stability and accuracy compared with existing solutions.

### 3.4. 3D Measurement Results

**Figure 6:**
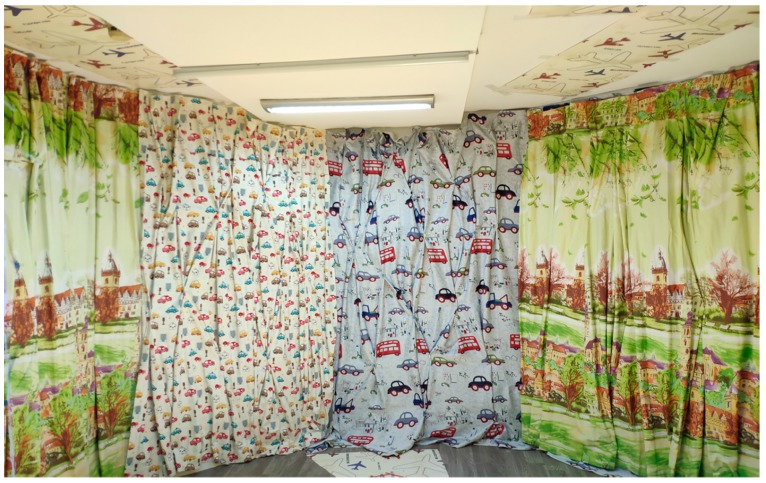
The real scene.

**Figure 7:**
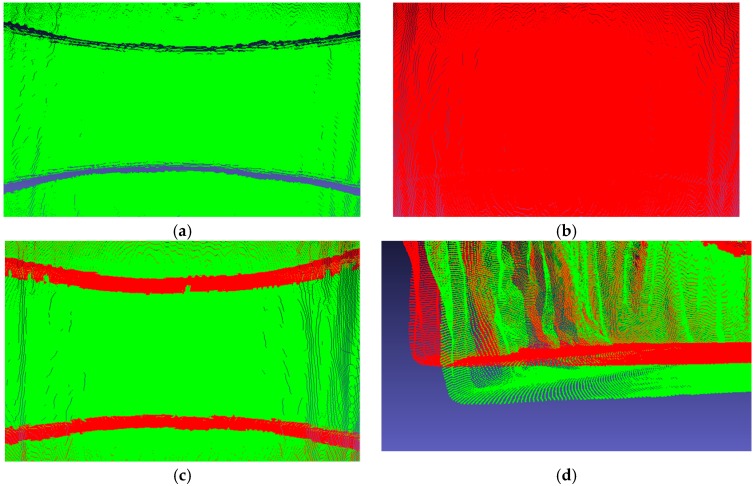
Point cloud fusion results of real scene. Red point clouds are the result of our method. Green point clouds are the result of method C. (**a**) and (**b**) are the results of method C and our method, respectively. (**c**) is the front of the real scene. (**d**) is the ground of the real scene.

In order to verify the effectiveness of the 3D results in practical applications, the multiple depth cameras calibration data obtained using method C and our method were applied to the point cloud fusion of the scanned clouds. Method C was chosen for this comparison because it is the best-performing of the three compared methods. We scanned the real scene of Figure 6 using the multiple depth camera system. The results of the point cloud fusion are shown in Figure 7.

It is evident that the result of point cloud fusion using the calibration result obtained by method C is not ideal. The point cloud scanned using each of the two depth cameras systems cannot be perfectly fused, and the cracks are large. This shows that the external parameters of the calibrated depth cameras are inaccurate, and there is considerable discrepancy compared with the actual values. Conversely, there are no cracks between the point clouds when using the calibration result obtained by our method and better fusion is achieved, which means that the actual values of external parameters between the depth cameras used are very close. As shown in Figure 7d, the results obtained using the two methods are quite different. Using Equations (1)–(6), we calculated the angle between the actual ground and the ground obtained by two methods. The angle value of method C was 3.588°, while the angle of our method is 0.342°, which means that the point cloud fusion results obtained using method C were distorted greatly by the ground calculations, while our method is more accurate. This experiment shows that the calibration method using the 3D target and the multiple depth camera systems proposed in this paper has very high accuracy.

**Figure 8:**
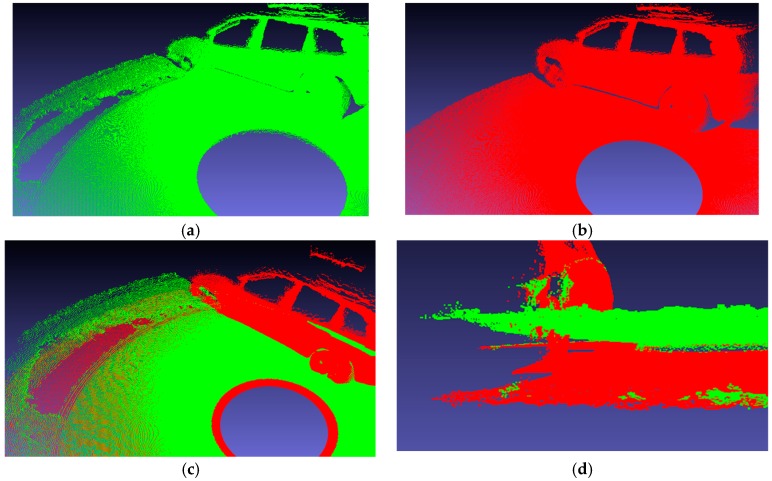
Point cloud fusion results of car model. Red point clouds are the result of our method. Green point clouds are the result of method C. (**a**) and (**b**) are the results of method C and our method, respectively. (**c**) is the front of the porcelain car model. (**d**) is the ground of the car model.

In addition, we used the same system to measure a car model at an auto show. Comparing the results of the point cloud fusion in Figure 8, the advantages of the calibration method presented in this paper are outstanding. The results obtained by method C show that there are huge cracks and unevenness on the ground, while the top of the vehicle model is distorted. In Figure 8d, the angle obtained using method C is 4.855°, and the angle of our method is 0.219°. The actual height and length of the car obtained from the official website are 163.50 cm and 441.50 cm respectively. The measured values by the two methods are shown in Table 7.

In contrast to method C, our method yields a flat ground and a vehicle model very close to the object. When applied to a large-scale model, the error of method C is greater, and our model gives better stability and accuracy.

**Figure 9:**
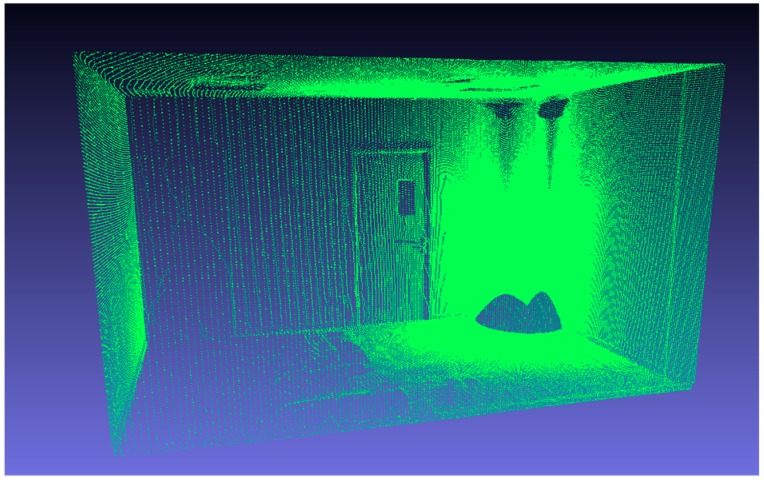
The model scanned by Faro.

In order to further compare the accuracy of method C and D with the proposed method, a closed regular room was used as a calibration object. The room was scanned by a Faro Focus 3D X330 lidar, as shown in Figure 9. The obtained Width, Height and Length are 243.80, 263.59, and 421.45 cm, regarded as the ground truth. The room was also measured by the three methods, as listed in Table 8.

The experimental results show that the proposed method is able to significantly improve the calibration accuracy of the method C. The calibration accuracy of our method is below 3 cm in the range of 4m. To summary, all the test results show that our calibrated system has good stability and accuracy in measuring complex three-dimensional indoor scenes.

## 4. Conclusions

In this paper, a new calibration method of the internal and external parameters for multiple depth cameras is proposed. Through a simple 3D target, multiple depth camera coordinates can be unified into the same world coordinate system, and the spatial–location relationship between them can be calculated. This method solves the relative orientation calibration of multiple depth cameras with limited or without overlapping fields of view. The accuracy and stability of the calibration are improved by precise calibration using the 3D target, shielding the light of the infrared projector and enhancing ambient brightness. In summary, compared with existing methods, the proposed method has the following advantages:

1. The proposed method calibrates through a 3D target using points obtained by lidar scanning. This allows calibration using a 3D target with minimal error, which can reduce the error caused by the subsequent calibration process from the source effectively.
2. All depth cameras can be calibrated simultaneously without a priori information on the cameras’ system parameters. This method has solved the problem of the global calibration of multiple cameras and the whole calibration process is simple and easy to apply.
3. The calibration of the depth cameras is simple and effective with good stability and high accuracy. There are no complex calculations, and the solving process is convenient and the calculation is fast.
4. The joint calibration process of multiple depth cameras is flexible and accurate. The efficiency achieved using the 3D target is much higher than that of plane calibration targets.

## Figures and Tables

**Table 1:** External parameters of each calibration board.

Calibration Boards	R	T
Left	$\left[\begin{array}{ccc} 0.7353 & -0.0245 & -0.6773\\ -0.0245 & 0.9977 & -0.0627\\ 0.6773 & 0.0627 & 0.7311 \end{array}\right]$	${\left[\begin{array}{ccc} -0.0691 & 0.1061 & -0.5436 \end{array}\right]}^{T}$
Mid	$\left[\begin{array}{ccc} 0.9998 & -0.0006 & -0.0187\\ -0.0006 & 0.9979 & -0.0647\\ 0.0187 & 0.0647 & 0.9977 \end{array}\right]$	${\left[\begin{array}{ccc} -0.0043 & 0.1132 & -0.5026 \end{array}\right]}^{T}$
Right	$\left[\begin{array}{ccc} 0.7575 & 0.0148 & 0.6527\\ 0.0148 & 0.9991 & -0.0400\\ -0.6527 & 0.0400 & 0.7566 \end{array}\right]$	${\left[\begin{array}{ccc} 0.0646 & 0.1100 & -0.5026 \end{array}\right]}^{T}$

**Table 2:** Intrinsic parameters of each camera.

Camera Name	$f_{x}$	$f_{y}$	$u_{0}$	$v_{0}$
Camera_up	577.38	578.35	320.02	253.88
Camera_mid	577.77	579.45	320.83	258.64
Camera_down	578.94	578.12	325.69	246.36

**Table 3:** External parameters between each camera.

Camera Set	R	T	Angle
Camera_up to camera_mid	$\left[\begin{array}{ccc} 1.0000 & -0.0017 & 0.0074\\ 0.0061 & 0.7404 & -0.6722\\ -0.0044 & 0.6722 & 0.7404 \end{array}\right]$	${\left[\begin{array}{ccc} -0.0008 & 0.5071 & 0.2918 \end{array}\right]}^{T}$	42.238°
Camera_down to camera_mid	$\left[\begin{array}{ccc} 0.9999 & -0.0091 & 0.0085\\ 0.0011 & 0.7440 & 0.6682\\ -0.0124 & -0.6682 & 0.7440 \end{array}\right]$	${\left[\begin{array}{ccc} -0.0055 & -0.5211 & 0.1340 \end{array}\right]}^{T}$	−41.926°

**Table 4:** Normal vectors of plane obtained by the three calibration methods.

Calibration Methods	Normal Vector of Ideal Plane	Normal Vector of Actual Plane	Included Angle
Method C	[0,0,1]	[−0.0451,−0.2547,0.9660]	14.9902°
Method D	[0,0,1]	[−0.0424,−0.0311,0.9986]	3.0142°
Our method	[0,0,1]	[0.0187,0.0247,0.9997]	1.7750°

**Table 5:** Angle between multiple depth cameras of the five calibration methods.

Camera Set	Ideal Angle	Method A	Method B	Method C	Method D	Our Method
Camera_up to camera_mid	42°	48.780°	44.103°	43.343°	42.850°	42.238°
Camera_down to camera_mid	−42°	−48.489°	−40.890°	−41.250°	−41.451°	−41.926°

**Table 6:** The RMS value of reprojection error of the five calibration methods.

Calibration Method	RMS Value/Pixel
Method A	0.3547
Method B	0.3558
Method C	0.3321
Method D	0.2843
Our method	0.1241

**Table 7:** Car size comparison.

Calibration Method	Height/cm		Length/cm	
	Measured	Absolute Error	Measured	Absolute Error
Method C	156.75	6.75	435.12	6.38
Our method	165.81	2.31	444.15	2.65

**Table 8:** Room size comparison.

Calibration Method	Width/cm		Height/cm		Length/cm	
	Measured	Absolute Error	Measured	Absolute Error	Measured	Absolute Error
Method C	239.70	4.10	257.43	6.16	414.10	7.35
Method D	245.98	2.18	266.77	3.18	425.19	3.74
Our method	246.28	2.48	265.76	2.17	424.15	2.7
